# Supplementary material for: Glucose Tolerance-Improving Activity of Helichrysoside in Mice and Its Structural Requirements for Promoting Glucose and Lipid Metabolism
Source: Int J Mol Sci. 2019 Dec 14;20(24):6322. doi: 10.3390/ijms20246322 (PMC6941121; doi:10.3390/ijms20246322)
Supplement: Supplementary file 1 [file ijms-20-06322-s001.pdf]

Supplementary Materials: Glucose Tolerance-Improving Activity of Helichrysoside in Mice and its Structural Requirements for Promoting Glucose and Lipid Metabolism

Toshio Morikawa [1,2,†,*], Akifumi Nagatomo [1,†], Takahiro Oka [1], Yoshinobu Miki [1], Norihisa Taira [1], Megumi Shibano-Kitahara [1], Yuichiro Hori [1], Osamu Muraoka [1,2] and Kiyofumi Ninomiya [1,2]

[1] Pharmaceutical Research and Technology Institute, Kindai University, 3-4-1 Kowakae, Higashi-osaka, Osaka 577-8502, Japan
[2] Antiaging Center, Kindai University, 3-4-1 Kowakae, Higashi-osaka, Osaka 577-8502, Japan
† These authors contributed equally to this work.
* Correspondence: morikawa@kindai.ac.jp; Tel. +81-6-4307-4306; Fax: +81-6-6729-3577

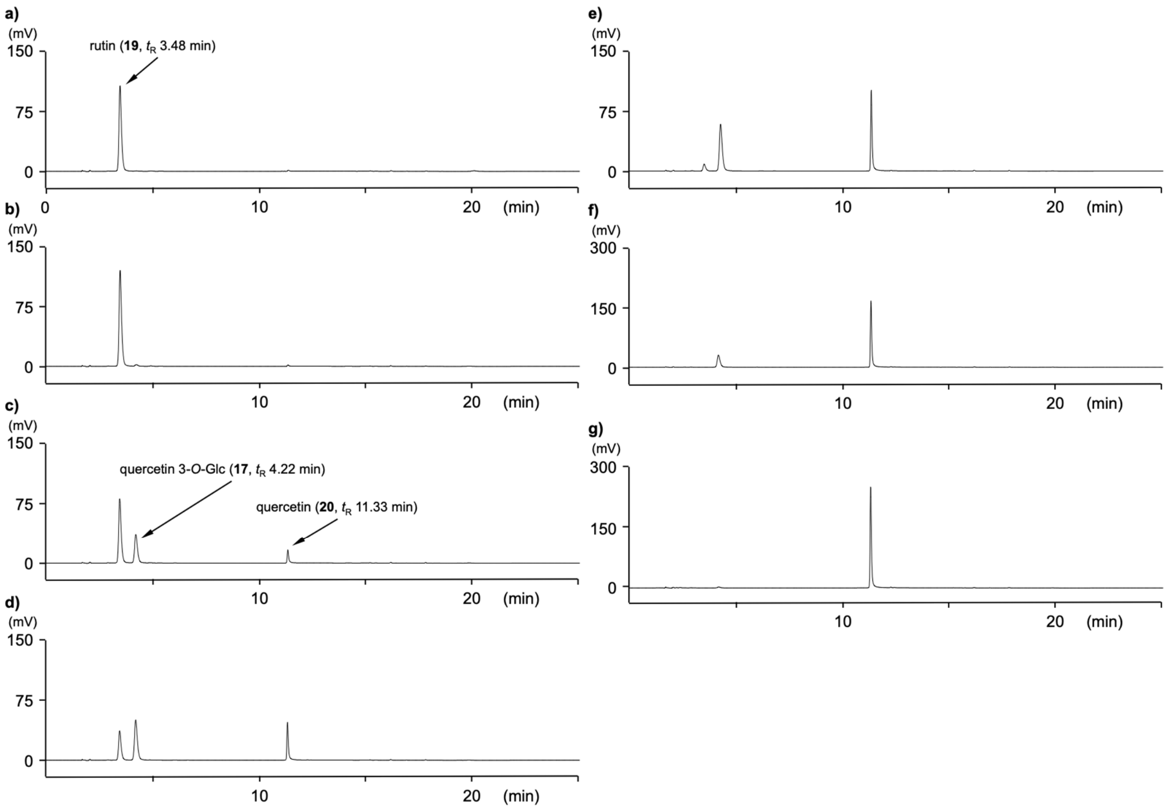

Figure S1. HPLC chromatograms (UV, 254 nm) of the reaction mixture after **a)** 0 min, **b)** 5 min, **c)** 30 min, **d)** 1 h, **e)** 2 h, **f)** 4.5 h, and **g)** 24 h.

Table S1. Linearities for rutin (**19**), quercetin 3-*O*-β–D-glucopyranoside (**17**), and quercetin (**20**).

Analyte	Regression Equation [a]	Correlation Coefficient (R^2)
Rutin (**5**)	$y = 6279.4\,x - 1830.5$	0.99995
Quercetin 3-*O*-β-D-glucopyranoside (**17**)	$y = 8747.4\,x - 11811$	0.99995
Quercetin (**20**)	$y = 14320\,x - 17934$	0.99997

[a] In the regression equation, *x* is the concentration of the analyte solution (μg/mL), and *y* is the peak area of the analyte.

Table S2. ^{13}C NMR data (150 MHz) for acylated flavonol glycosides (**2**–**6**, **9**, **11**, **13**–**15**).

Position	**2**[a]	**3**[b]	**4**[b]	**5**[b]	**6**[b]	**9**[b]	**11**[b]	**13**[b]	**14**[b]	**15**[b]
2	159.3	156.8	156.36	156.4	156.4	156.3	156.5	156.5	156.2	156.3
3	135.3	132.1	133.3	133.3	133.3	133.1	133.2	133.3	133.0	133.2
4	179.4	177.9	177.5	177.5	177.5	177.3	177.5	177.5	177.3	177.4
5	163.1	161.7	161.2	161.3	161.3	161.2	161.4	161.4	161.1	161.2
6	100.0	99.3	98.9	98.8	99.0	98.6	98.9	98.8	98.7	98.7
7	166.2	164.9	164.4	164.1	164.6	164.0	164.6	164.3	164.3	164.2
8	94.8	94.0	93.6	93.5	93.7	93.4	93.7	93.7	93.2	93.4
9	158.5	161.7	156.39	158.0	156.5	156.3	156.5	156.6	156.2	156.4
10	105.6	104.4	103.9	104.0	103.9	103.9	104.0	104.1	103.7	103.8
1'	123.1	127.0	121.2	122.3	121.2	121.0	121.2	121.3	121.0	121.2
2'	117.3	116.8	116.3	115.3	116.3	116.1	116.3	116.3	116.0	115.2
3'	145.9	133.9	145.0	144.9	145.0	144.7	145.0	145.0	144.8	144.9
4'	149.8	145.4	148.6	148.6	148.7	148.4	148.7	148.6	148.4	148.5
5'	115.9	115.7	115.4	111.8	115.3	115.1	115.3	115.3	115.1	116.1
6'	123.4	122.0	121.6	121.6	121.6	121.4	121.6	121.7	121.4	121.6
Glc-1''	104.0	101.3	101.0	100.9	101.1	100.8	100.9	101.1	100.7	101.0
2''	75.6	74.6	74.2	74.2	74.2	74.0	74.2	74.1	73.9	74.1
3''	78.1	77.0	76.5	76.5	76.5	76.3	76.5	76.5	76.4	76.5
4''	71.7	70.7	70.1	70.3	70.1	69.9	70.1	70.2	70.0	70.5
5''	75.6	74.9	74.4	74.4	74.4	74.0	74.3	74.1	74.4	74.4
6''	64.1	63.8	63.3	63.4	63.5	62.8	63.0	63.2	63.4	64.0
Acyl-1'''	127.6	127.0	120.8	121.3	135.2	125.8	125.9	134.6	120.4	124.6
2'''	133.7	130.4	156.9	156.4	114.7	117.7	114.6	128.0	112.4	106.5
3'''	115.7	114.9	116.3	116.3	157.8	144.4	146.9	129.0	147.2	141.9
4'''	159.9	149.0	131.6	132.0	117.8	147.2	148.6	129.7	151.3	152.6
5'''	115.7	114.9	119.5	120.8	130.0	114.8	115.0	129.0	115.0	141.9
6'''	133.7	130.4	128.7	128.6	119.2	123.5	125.5	128.0	123.0	106.5
7'''	145.3	144.6	140.3	139.4	144.7	143.7	144.1	142.6	165.2	164.9
8'''	116.2	115.4	116.6	117.6	117.4	114.4	114.7	119.0		
9'''	167.8	166.5	166.5	166.3	165.9	165.3	165.7	165.3		
3'''-OCH_3		55.9		55.7			55.6		55.4	55.9
4'''-OCH_3										60.2

Measured in [a]CD_3OD and [b]DMSO-*d*$_6$

Compound names: quercetin 3-*O*-(6''-*O*-*cis*-*p*-methylcoumaroyl)-β-D-glucopyranoside (**2**), quercetin 3-*O*-(6''-*O*-*trans*-*p*-methylcoumaroyl)-β-D-glucopyranoside (**3**), quercetin 3-*O*-(6''-*O*-*trans*-*o*-coumaroyl)-β-D-glucopyranoside (**4**), quercetin 3-*O*-(6''-*O*-*trans*-*o*-methylcoumaroyl)-β-D-glucopyranoside (**5**), quercetin 3-*O*-(6''-*O*-*trans*-*m*-coumaroyl)-β-D-glucopyranoside (**6**), quercetin 3-*O*-(6''-*O*-*cis*-caffeoyl)-β-D-glucopyranoside (**9**), quercetin 3-*O*-(6''-*O*-*cis*-feruloyl)-β-D-glucopyranoside (**11**), quercetin 3-*O*-(6''-*O*-*cis*-cinnamoyl)-β-D-glucopyranoside (**13**), quercetin 3-*O*-(6''-*O*-vanilloyl)-β-D-glucopyranoside (**14**), and quercetin 3-*O*-(6''-*O*-trimethylgalloyl)-β-D-glucopyranoside (**15**).

Table S3. Effects of helichrysoside (**1**) on glucose tolerance test after 14 days administration in mice.

Treatment	Dose (mg/kg/day, *p.o.*)	N	Boold Glucose (mg/dL)				AUC
			0 min	30 min	60 min	120 min	(h·mg/dL)
Control	—	11	117.2 ± 4.0	324.0 ± 9.2	254.9 ± 13.2	169.5 ± 7.7	467.3 ± 16.0
Helichrysoside (**1**)	1	6	105.9 ± 5.1	313.8 ± 18.9	209.1 ± 15.8 *	159.5 ± 7.8	419.9 ± 24.1
	10	6	107.3 ± 5.0	290.1 ± 13.6	206.7 ± 9.9 *	149.6 ± 9.1	401.7 ± 16.9 *

Each value represents the mean ± S.E.; asterisks denote significant differences from the control group, * $p < 0.05$.

Table S4. Effects of helichrysoside (**1**) on food intake, visceral fat weight, liver weight, liver triglyceride content, and plasma parameters after 14 days administration in mice.

Treatment	Dose (mg/kg/day, p.o.)	N	Food Intake (g/mouse/day)	Epididymal Fat[a] (mg)	Mesenteric Fat[b] (mg)	Paranephric Fat[c] (mg)	Visceral Fat[(a+b+c)] (mg)	Liver Weight (mg)	Liver Triglyceride (mg/g)	Plasma Triglyceride (mg/dL)	Plasma Cholesterol (mg/dL)	Plasma Free Fatty Acid (mEq/L)
Control	—	11	4.8 ± 0.2	1089 ± 104	758 ± 44	367 ± 34	2214 ± 172	1626 ± 56	36.6 ± 1.3	137 ± 7	122 ± 5	1.80 ± 0.05
Helichrysoside (**1**)	1	6	4.7 ± 0.2	923 ± 158	687 ± 98	338 ± 79	1948 ± 327	1588 ± 30	32.0 ± 1.2	127 ± 8	117 ± 4	1.74 ± 0.11
	10	6	5.0 ± 0.2	987 ± 98	717 ± 55	328 ± 57	2032 ± 206	1557 ± 79	33.1 ± 1.7	141 ± 15	119 ± 4	1.65 ± 0.17

Each value represents the mean ± S.E.; significant differences were not observed.
